# Combined effects of high atrial septal pacing and reactive atrial antitachycardia pacing for reducing atrial fibrillation in sick sinus syndrome

**DOI:** 10.1002/joa3.12888

**Published:** 2023-06-26

**Authors:** Hironobu Sumiyoshi, Hiroshi Tasaka, Kenta Yoshida, Mitsuru Yoshino, Kazushige Kadota

**Affiliations:** ^1^ Department of Cardiovascular Medicine Kurashiki Central Hospital Kurashiki Japan

**Keywords:** atrial fibrillation, high atrial septal pacing, reactive atrial antitachycardia pacing, sick sinus syndrome

## Abstract

**Background:**

It is unknown whether atrial fibrillation (AF) burden varies by pacing site in patients with reactive atrial antitachycardia pacing (rATP). We aimed to compare AF burden in patients with high atrial septal pacing (HASp) via delivery catheter and right atrial appendage pacing (RAAp) in patients with sick sinus syndrome (SSS).

**Methods:**

We retrospectively identified 109 patients with a history of paroxysmal AF and SSS who had received dual‐chamber pacemaker implantation between January 2017 and December 2019, of whom 39 and 70 patients had HASp and RAAp, respectively. rATP was initiated after a 1‐month post‐implantation run‐in period.

**Results:**

Patients with HASp had a significantly shorter P‐wave duration during atrial pacing than those with RAAp (99.3 ± 10.4 vs. 116.0 ± 14.3 ms, *p* < .001). During the 3‐year follow‐up period, the incidence of an AF lasting longer than 1 or 7 days was significantly lower (hazard ratio [HR], 0.45; *p* = .016; HR, 0.24; *p* = .004) than in those with RAAp. The median time of AF/AT per day in the follow‐up periods was significantly shorter in the HASp group than in the RAAp group (10 vs. 18 min/day, *p* = .018). Atrial lead division did not occur in the HASp group during the follow‐up period.

**Conclusions:**

HASp via delivery catheter is as safe as RAAp, and HASp combined with rATP is effective for reducing AF burden in patients with SSS and paroxysmal AF.

## Graphical abstract

High atrial septal pacing combined with reactive atrial antitachycardia pacing is effective for reducing atrial fibrillation (AF) burden in patients with sick sinus syndrome and paroxysmal AF.

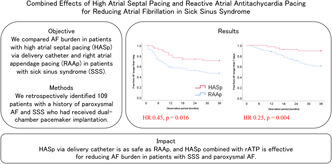

## 1 INTRODUCTION

Atrial fibrillation (AF) is a common arrhythmia in patients with cardiac implantable electronic devices (CIEDs). Some studies have reported that right atrial appendage pacing (RAAp) exacerbates interatrial conduction delay (IACD), whereas high atrial septal pacing (HASp) may decrease AF incidence and duration.^1^, ^2^, ^3^ However, other studies have shown that right atrial septal pacing alone did not suppress AF.^4^, ^5^ Previous methods of atrial lead implantation have used tined or screw leads. Lead implantation in patients with high atrial septum (HAS) is difficult using a screw lead alone because there is no pectinate muscle on the atrial septum. Therefore, RAAp remains the first‐line choice for atrial pacing sites.^6^ Recently, a delivery catheter method became available; HAS lead implantation is easy and accurate using this device.^7^

AF is irregular and is not susceptible to pace termination. However, when AF becomes slower, organized rhythms, including atrial flutter (AFL) or atrial tachycardia (AT), emerge, which can be terminated using antitachycardia pacing (ATP).^8^ Reactive atrial antitachycardia pacing (rATP) is a second‐generation iteration of atrial ATP and has been reported to suppress chronic AF development in patients with CIEDs and a history of paroxysmal or persistent AF/AT in a randomized multicenter international trial (MINERVA trial).^9^

Given that much of the substrate for atrial arrhythmias is known to be located in the left atrium (LA), HASp may improve rATP efficacy owing to the closer proximity of the pacing site to the reentrant circuit. Therefore, we hypothesized that accurate HASp combined with rATP is effective for reducing AF burden in patients with sick sinus syndrome (SSS) because HASp may reduce AF incidence. Furthermore, rATP is more effective in patients with HAS than in those with right atrial appendage (RAA).

## 2 METHODS

### 2.1 Study population

**FIGURE 1:**
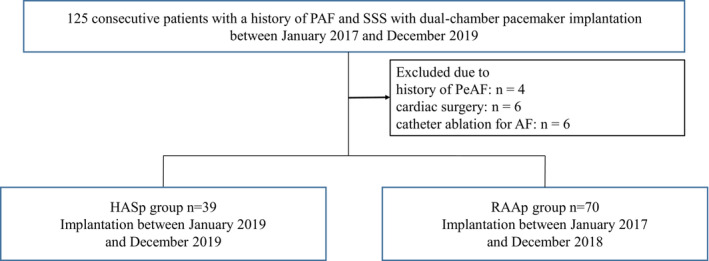
Study flow chart. HASp, high atrial septal pacing; PAF, paroxysmal atrial fibrillation; PeAF, persistent atrial fibrillation; RAAp, right atrial appendage pacing; SSS, sick sinus syndrome.

This was a retrospective, single‐center, observational study that was approved by Kurashiki Central Hospital Medical Ethics Committee and adhered to the Helsinki Declaration. Between January 2017 and December 2019, 125 consecutive patients with a history of paroxysmal atrial fibrillation (PAF) and SSS who had received dual‐chamber pacemaker implantation (Azure XT DR MRI, Medtronic Inc.) at Kurashiki Central Hospital were included in this study. rATP, atrial rate stabilization (ARS), and post‐mode switch overdrive pacing (PMOP) were started in all patients 1 month following pacemaker implantation. Patients with a history of cardiac surgery, a history of catheter ablation, and persistent AF for more than 7 days at the time of rATP initiation were excluded. As implantation of HASp was first performed in our hospital beginning in January 2019, all patients treated before this date were implanted with RAAp. After January 2019, all patients were implanted with HASp (Figure 1). Of 125 screened patients, 109 met the inclusion criteria. Of these, 39 and 70 patients were assigned to the HASp and RAAp groups, respectively. Patients underwent follow‐up examination in their respective therapy groups at 1 and 6 months after implantation and thereafter every 6 months until the 36th month after implantation.

### 2.2 Implantation procedure

**FIGURE 2:**
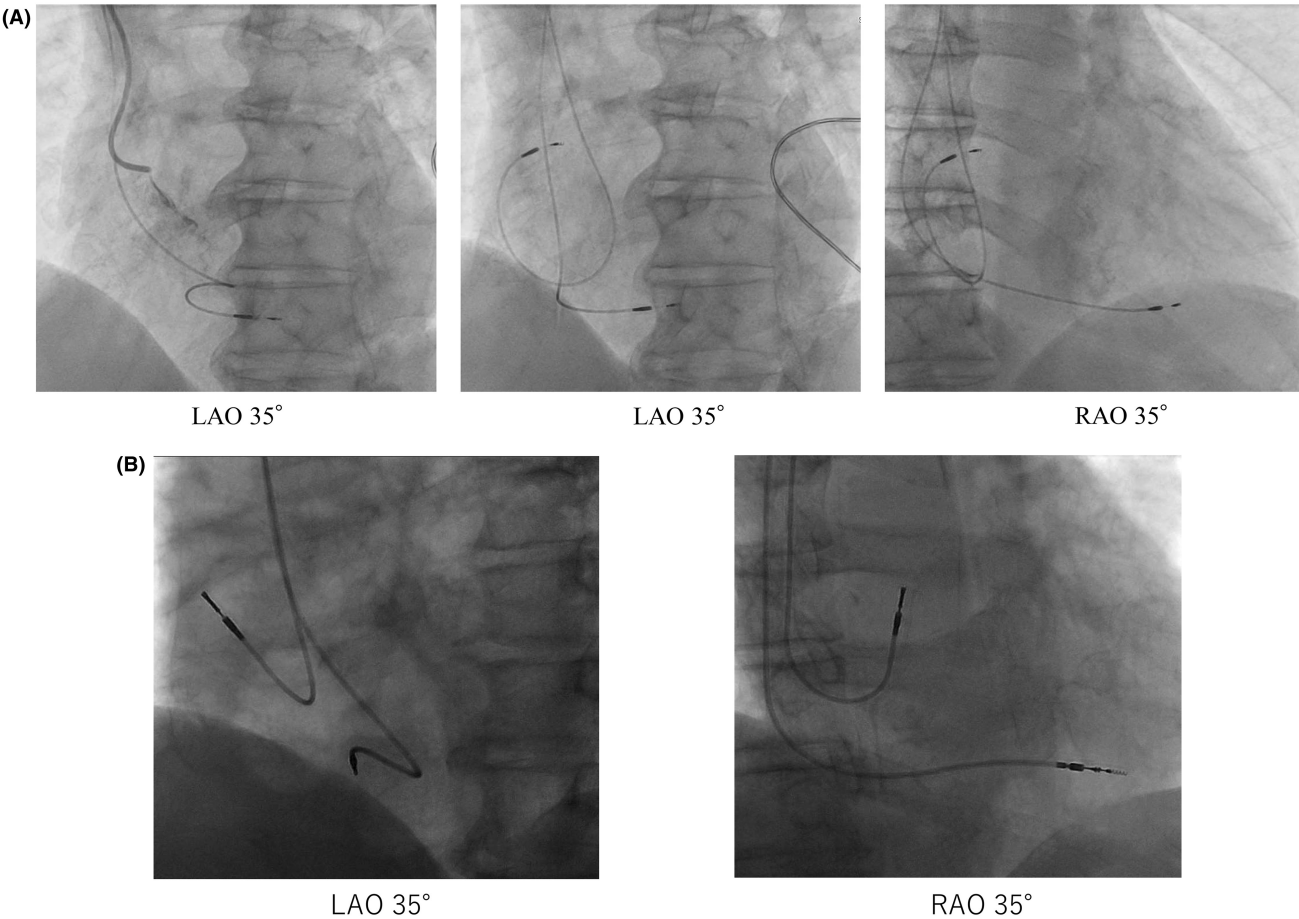
Atrial lead fluoroscopy for patients from each atrial pacing group. (A) Fluoroscopic views of HAS. (B) Fluoroscopic views of RAA. HAS, high atrial septum; LAO, left anterior oblique; RAA, right atrial appendage; RAO, right anterior oblique.

In patients with HAS, atrial lead position was determined using fluoroscopy; right atrium angiography was performed superior to the foramen ovale. A Medtronic Select Secure 3830 lead and a C315 Delivery Catheter (S4 or S5) were used to perform HASp. With the fluoroscope in the left anterior oblique position, the delivery catheter was rotated toward the interatrial septum. The catheter was withdrawn until the tip was straightened at the roof of the right atrium. The confluence of the atrial septum and the right atrial roof was confirmed in this area by injecting a contrast medium from the catheter. Subsequently, the fluoroscope was switched to the right anterior oblique view, and the catheter was positioned anteriorly. Finally, the lead was advanced through a delivery catheter, screwed toward the left side of the HAS, and lead sensing and pacing tests were performed (Figure 2A). The Medtronic 5076 lead or Medtronic 4574 lead was used to implant RAA leads using standard techniques (Figure 2B).

### 2.3 Mechanism of rATP

The rATP algorithm has been previously described in detail.^9^, ^10^, ^11^ rATP can terminate AF/AT rhythms when they spontaneously organize to AFL or AT, even in persistent episodes. When the device detects an AF/AT episode, it monitors rhythm transitions based on atrial cycle length (CL) regions and regularity; if the rhythm shifts to a different CL region owing to a change in CL or regularity, the device delivers ATP therapy again in the new region. Up to three ATP therapies (Ramp or Burst+) are delivered for each region to which the episode may transition. Successful therapy was defined as sinus rhythm recovery within 20 s of the last sequence during an episode. The success rate was calculated as the number of pacing‐terminated episodes divided by the total number of episodes that rATP attempted to terminate. Although ATP cannot terminate true AF, this algorithm can redeliver ATP when it detects a change in the patient's rhythm such that the rhythm may be more vulnerable to termination with ATP.

### 2.4 Data collection and outcomes

We assessed the outcomes of AF/AT and rATP treatment using a remote monitoring system every month as well as through device interrogation every 5–7 months in the outpatient clinic. Clinical data, including age, sex, comorbidities, echocardiographic data, and medications, were assessed from the medical records. Moreover, data regarding atrial lead performance and atrial intervention pacing functions (ARS, PMOP) were collected. The longest P‐wave duration in the inferior leads (II, III, and aVF) was measured using a standard surface 12‐lead electrocardiogram at the pacemaker implantation day. To verify diagnostic accuracy and rule out far‐field R‐wave sensing, atrial lead oversensing, pacemaker‐mediated arrhythmia, and/or electrical/mechanical interference, AF/AT episodes were confirmed by a clinician. AF/AT burden was derived from pacemaker counters. The mean percentage of pacing and AF/AT duration/day were calculated in overall observation periods.

### 2.5 Statistical analysis

Baseline characteristics were compared between patients who underwent HASp and RAAp. Continuous data were presented as means and standard deviations or medians and interquartile ranges, as appropriate. Categorical data were expressed as counts and percentages. Comparisons of clinical characteristics between the two groups were performed using Student's *t*‐test. The Mann–Whitney *U*‐test and chi‐squared test or Fisher's exact test were used for continuous and categorical data, respectively. The analyses of the time to the first AF/AT episode lasting ≥1, ≥7, and ≥30 days were described using the Kaplan–Meier method; survival curves were compared using a log‐rank test. To compare AF/AT‐free survival between the two groups, univariate Cox proportional hazards regression was used. All statistical analyses were two‐sided, and *p*‐values <.05 were considered statistically significant. All statistical analyses were performed using Statistical Package for the Social Sciences software version 25 (SPSS Inc.).

## 3 RESULTS

### 3.1 Baseline characteristics

The median follow‐up was 36 (range, 12–36) months. Baseline characteristics at the time of pacemaker implantation are shown in Table 1. The mean lead impedance, pacing, and sensing thresholds at the last available follow‐up are presented in Table 2. No significant differences were observed in clinical characteristics, including age, male sex, comorbidities, echocardiographic features, and medications, between the two groups.

**TABLE 1:** Clinical characteristics of patients at the time of pacemaker implant.

	HASp (*n* = 39)	RAAp (*n* = 70)	*p*‐value
Age, mean ± SD	81.6 ± 6.3	80.3 ± 6.7	.313
Sex, male, *n* (%)	19 (48.7)	34 (48.6)	.988
Body mass index, mean ± SD	22.4 ± 3.2	22.8 ± 3.2	.508
Comorbidities			
History of CHF, *n* (%)	7 (17.9)	8 (11.4)	.344
Hypertension, *n* (%)	31 (79.5)	47 (67.1)	.171
Diabetes, *n* (%)	12 (30.8)	19 (27.1)	.687
History of stroke, *n* (%)	4 (10.3)	10 (14.3)	.547
Medications			
AAD, *n* (%)	4 (10.3)	11 (15.7)	.428
β‐adrenergic blocker, *n* (%)	18 (46.2)	32 (45.7)	.965
ACEi or ARB, *n* (%)	19 (48.7)	27 (38.6)	.304
Pacing characteristics			
Lower rate limit (bpm), mean ± SD	60.5 ± 2.2	60.2 ± 1.8	.448
Upper rate (bpm), mean ± SD	123.6 ± 4.9	122.9 ± 4.9	.452
ARS, *n* (%)	39 (100)	70 (100)	
APP, *n* (%)	0	0	
PMOP, *n* (%)	39 (100)	70 (100)	
MVP, *n* (%)	39 (100)	70 (100)	
Echocardiographic parameters			
Left atrial diameter (mm), mean ± SD	39.2 ± 4.9	39.4 ± 6.4	.886
Left atrial volume index, mean ± SD	49.6 ± 19.2	47.0 ± 16.5	.467
Ejection fraction, mean ± SD	58.9 ± 8.4	58.7 ± 7.2	.881
P‐wave characteristics			
Sinus P‐wave duration (ms), mean ± SD	107.3 ± 11.9	107.6 ± 15.1	.936
Paced P‐wave duration (ms), mean ± SD	99.3 ± 10.4	116.0 ± 14.3	<.001
Operation time	99.8 ± 35.5	87.9 ± 36.9	.105

Abbreviations: AAD, antiarrhythmic drugs; ACEi, angiotensin‐converting enzyme inhibitor; APP, atrial preference pacing; ARB, angiotensin receptor blocker; ARS, atrial rate stabilization; HASp, high atrial septal pacing; MVP, managed ventricular pacing; PMOP, post‐mode switch overdrive pacing; RAAp, right atrial appendage pacing; SD, standard deviation.

**TABLE 2:** Pacing characteristics and complications following long‐term follow‐up.

	HASp (*n* = 39)	RAAp (*n* = 70)	*p*‐value
Atrial lead parameters at last follow‐up			
Overall follow‐up (months), mean ± SD	36.0 (23.0–36.0)	36.0 (24.0–36.0)	.507
Threshold (V@0.4 ms), mean ± SD	0.70 ± 0.32	0.71 ± 0.29	.856
P‐wave sensing threshold (mV), mean ± SD	1.70 ± 0.64	1.96 ± 1.1	.164
Impedance (Ω), mean ± SD	531 ± 83	477 ± 67	<.001
Atrial pacing % of time (25th–75th percentile)	70.6 (43.3–82.8)	72.0 (50.1–91.3)	.254
Atrial lead complications			
Atrial lead revisions, *n* (%)	0 (0)	1 (1.4)	.453
Ventricular pacing % of time (25th–75th percentile)	3.0 (1.0–5.0)	3.0 (0.7–10.5)	.784
AF/AT per day (min)	10.0 (3.0–18.0)	18.0 (4.0–108.0)	.018

Abbreviations: AF, atrial fibrillation; AT, atrial tachycardia; HASp, high atrial septal pacing; RAAp, right atrial appendage pacing; SD, standard deviation.

### 3.2 Implantation time, safety, and P‐wave characteristics

No significant differences were noted between patients in the HASp and RAAp groups regarding surgical duration or atrial lead revision. Furthermore, the mean pacing and sensing thresholds at the last available follow‐up were not significantly different. The baseline sinus P‐wave duration was not significantly different between the two groups (107.3 vs. 107.6 ms, *p* = .936); however, the HASp group had a significantly shorter paced P‐wave duration than the RAAp group (99.3 vs. 116.0 ms, *p* = .001).

### 3.3 rATP efficacy

A total of 49 patients (15 [38.5%] and 34 [48.6%] in the HASp and RAAp groups, respectively) experienced more than ten rATP therapies during the follow‐up periods. The baseline characteristics of the patients who experienced rATP therapy were compared between the HASp and RAAp groups (Table S1). No significant differences were observed in clinical characteristics, including age, male sex, comorbidities, echocardiographic features, and medications, between the two groups. A total of 45 231 rATP‐treated episodes in 49 patients were evaluated. The median number of rATP therapies was 98 (27–632) per patient. The overall median rATP success rate was 38.9% (19.8%–51.2%). No significant difference was observed between the groups in the median number of rATP therapies (107 vs. 91, *p* = .894). Patients with HASp had a significantly higher success rate of rATP than those with RAAp (47.8% vs. 27.0%, *p* = .033) (Table 3).

**TABLE 3:** rATP characteristics between the HASp and RAAp groups.

	HASp	RAAp	*p*‐value
rATP experienced patients, *n* (%)	15 (38.5)	34 (48.6)	.309
Total rATP therapies	10 248	34 983	
Number of rATP therapies per patient (25th–75th percentile)	107 (18–717)	97 (32–536)	.828
Total rATP success	5603	14 658	
Total rATP success/Total rATP therapies (%)	54.70	41.9	<.001
Median rATP success rate (25th–75th percentile)	50.0 (38.9–62.0)	32.7 (15.7–47.0)	.016

Abbreviations: HASp, high atrial septal pacing; RAAp, right atrial appendage pacing; rATP, reactive atrial antitachycardia pacing.

### 3.4 AF/AT‐free survival

**FIGURE 3:**
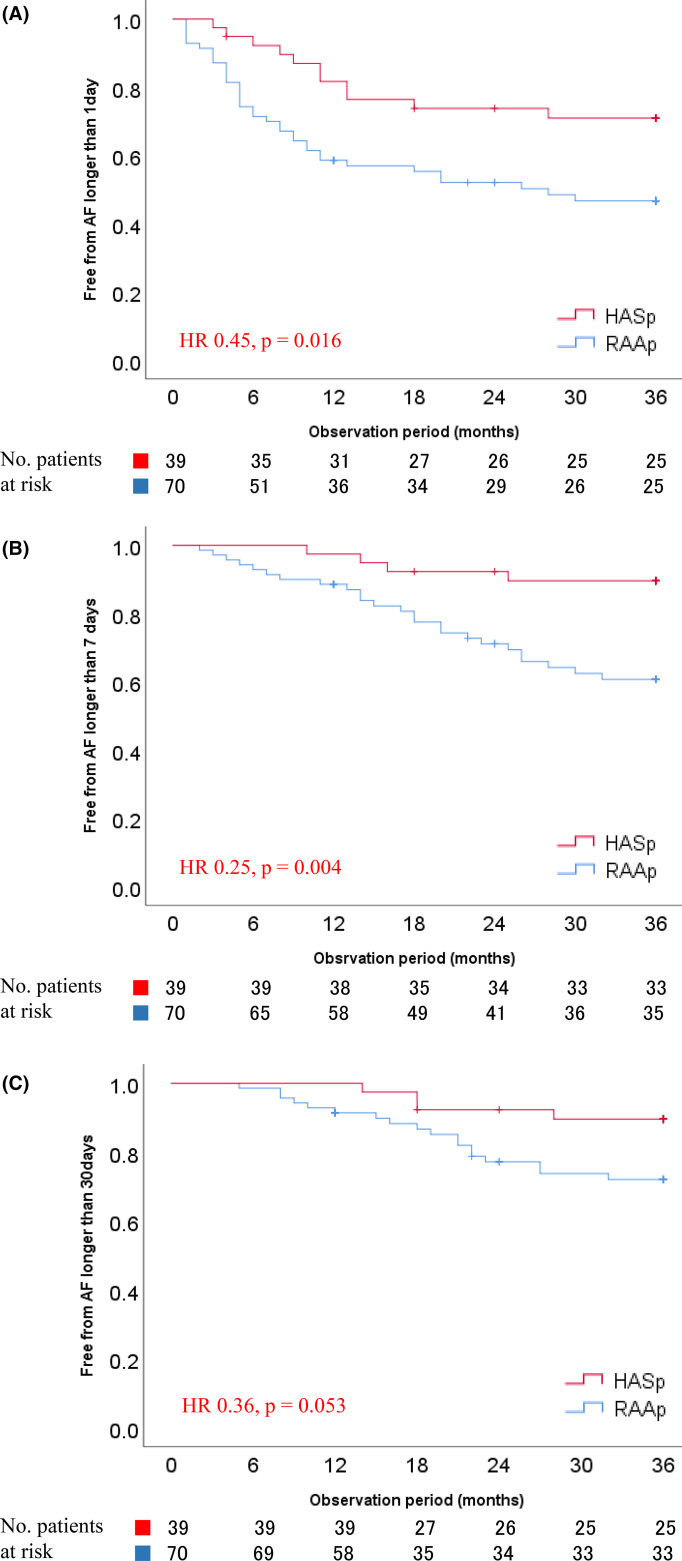
Comparisons of the Kaplan–Meier survival curves of AF‐free episodes lasting ≥1 day (A), ≥7 days (B), and ≥ 30 days (C). AF, atrial fibrillation; HASp, high atrial septal pacing; RAAp, right atrial appendage pacing; rATP, reactive atrial antitachycardia pacing.

The HASp group had a significantly lower incidence of AF/AT lasting ≥1 day (hazard ratio [HR], 0.45; 95% confidence interval [CI], 0.23–0.89; *p* = .016) and ≥7 days (HR, 0.25; 95% CI, 0.09–0.70; *p* = .004) than the RAAp group. The incidence of an AF/AT lasting ≥30 days was not significantly different between the two groups (HR, 0.36; 95% CI, 0.12–1.1; *p* = .053) (Figure 3).

Subgroup analyses were conducted to assess the effect of atrial pacing (Ap) on the risk of AF/AT. Table S2 compares the baseline characteristics between HASp and RAAp in the high percentage (≥75%; *n* = 50) of Ap group. Table S3 compares the baseline characteristics between HASp and RAAp in the low percentage (<75%; *n* = 59) of Ap group. In patients with high percentage of Ap, those with HASp had a significantly lower incidence of AF/AT lasting ≥1 day (HR, 0.67; 95% CI, 0.44–0.98; *p* = .027) and ≥7 days (HR, 0.26; 95% CI, 0.04–0.97; *p* = .015) than those with RAAp. AF/AT lasting ≥30 days (HR, 0.29; 95% CI, 0.21–2.81; *p* = .068) were not significantly different between the two groups (Figure S1). In patients with low percentage of Ap, AF/AT lasting ≥1 day (HR, 0.74; 95% CI, 0.51–1.06; *p* = .224), ≥7 days (HR, 0.63; 95% CI, 0.45–0.90; *p* = .088) and ≥ 30 days (HR, 0.82; 95% CI, 0.57–1.19; *p* = .283) were not significantly different between the two groups (Figure S2).

Another subgroup analysis was conducted to assess the effect of Ap on the risk of AF/AT. In patients who experienced rATP therapy, those with HASp had a significantly lower incidence of AF/AT lasting ≥7 days (HR, 0.63; 95% CI, 0.45–0.90; *p* = .006) than those with RAAp. AF/AT lasting ≥1 day (HR, 0.79; 95% CI, 0.62–1.02; *p* = .053) and ≥30 days (HR, 0.75; 95% CI, 0.52–1.07; *p* = .098) were not significantly different between the two groups (Figure S3).

### 3.5 AF/AT burden

The median time of AF/AT per day in the follow‐up periods was significantly shorter in the HASp group than in the RAAp group (10 vs. 18 min/day, *p* = .018) (Table 2).

## 4 DISCUSSION

AF accounts for approximately 40%–70% of patients with sinus node dysfunction.^12^ The major finding of this retrospective analysis is that among patients with PAF and SSS, HASp using a delivery catheter and rATP was associated with decreased AF burden compared with RAAp with rATP. This may be because HASp shortens interatrial conduction, which can suppress PAF incidence. Reducing IACD and modifying dispersion of atrial refractoriness are mechanisms by which HASp has been theorized to reduce AF compared with RAAp.^2^, ^13^ In this study, the mean paced P‐wave difference between the two groups was 16.7 ms. We experienced a representative case that total atrial activation time during HASp was shorter than that of RAAp before catheter ablation for AF in the context of SSS after follow‐up periods. In that case, the conduction time from pacing to most delayed activation site in the lateral side of LA was 30 ms faster from HAS than that from RAA (Figure S4).

Furthermore, another subset analysis showed that patients who experienced rATP therapy in the HASp group experienced a decreased number of AF/AT lasting ≥7 days. AF typically originates from the pulmonary veins and LA. Some studies have shown that the dominant frequency of AF is generally higher in the LA than that in the RA.^14^, ^15^ In the RAAp group, CL pacing by rATP is determined by the AF/AT frequency sensed in the RA. Effective pacing stimulation may not be successfully achieved when the LA CL is different from the RA CL and the pacing site is far from the reentrant circuit. Owing to the closer proximity of the pacing site to the reentrant circuit, HASp may improve rATP efficacy. Although rATP cannot terminate true AF, it can redeliver ATP when it detects a change in the rhythm of AF/AT to a moment when the rhythm may be more vulnerable to termination with ATP. Therefore, rATP cannot reduce PAF incidence but can reduce AF/AT lasting ≥7 days. Conversely, the incidence of AF/AT‐free periods lasting ≥30 days was not significantly different between the two groups. Previous studies have demonstrated spatiotemporal organization in PAF, with a left‐to‐right atrial frequency gradient during AF; however, the LA‐to‐RA frequency was attenuated in patients with long‐lasting (>1 month) persistent AF.^16^ Therefore, HAS CL and RAA CL of AF/AT may be the same in patients with long‐lasting persistent AF. Furthermore, atrial pacing is not operated during AF/AT, although rATP is operated at that time. This may explain why the number of AF/AT lasting ≥30 days was not significantly different between the pacing sites.

Although HASp is believed to improve interatrial conduction and reduce AF burden, HASp alone has failed to show consistent benefits.^4^, ^5^ One reason may be that the area of the HAS is difficult to implant using conventional tools. There is no pectinate muscle on the HAS, unlike the RAA, and conventional leads may slip and fail to insert into the HAS. To date, two types of delivery systems are available for HASp. The first is a specially shaped stylet, and the second is a delivery catheter.^7^, ^17^ We believe that the delivery catheter is more useful than the stylet because the lead can insert more deeply toward the LA with delivery catheter support. Furthermore, the HAS can be confirmed before insertion by injecting a contrast medium via the delivery catheter. In our study, HASp using a delivery catheter was as safe as RAAp. Although HASp had a longer mean surgical implantation time than RAAp, this can be improved with experience. HASp combined with rATP may have a synergistic effect in reducing AF burden in patients with SSS.

### 4.1 Study limitations

Our study had some limitations. First, this was a small, single‐center, retrospective study and was not randomized. The small number of patients may limit the interpretation of the results. Although we cannot rule out potential bias using our data, baseline characteristics were almost identical between the two groups. Second, the atrial lead position was determined using fluoroscopy, and the right atrium was identified using angiography. Although we used a delivery catheter for accurate atrial septum implantation in the HASp procedure, there may be low atrial septal pacing in the HASp group. As a conduction system pacing mechanism, fluoroscopically and electrically defined HASp may be more beneficial as an alternative to RAAp. Third, the definition of a successful rATP was termination within 20 s following the last sequence. Therefore, AF/AT may have recurred immediately after the last rATP delivery, and frequent rATP therapies may have been required in some cases. Fourth, not only rATP but ARS and PMOP were also used in this study. These atrial preventive pacing methods are also effective for reducing AF burden.^9^ Lastly, this study included patients with a history of PAF and SSS. The LA‐to‐RA frequency is attenuated in patients with long‐lasting (>1 month) persistent AF.^14^ The HAS CL and RAA CL of AF/AT may be the same in patients with long‐lasting persistent AF. Furthermore, atrial pacing is not performed during AF/AT, although rATP is operated at that time. This may explain why the number of AF/AT lasting ≥30 days was not significantly different between the pacing sites. Therefore, the advantage of HASp may be different in patients with persistent AF. Moreover, we did not include patients with atrioventricular block. Low atrial septum pacing (LASp) may be more beneficial for these patients as LASp can shorten atrioventricular conduction and reduce ventricular pacing.

## 5 CONCLUSIONS

In conclusion, HASp using a delivery catheter is as safe as RAAp. HASp may improve IACD and the success rate of rATP. Therefore, the combination of HASp and rATP may have a synergistic effect in reducing AF in SSS. Further prospective, multicenter studies are warranted to confirm these findings.

## CONFLICT OF INTEREST STATEMENT

The authors declare no conflict of interests for this article.

## ETHICS STATEMENT

This study was approved by Kurashiki Central Hospital Medical Ethics Committee (No. 4086).

## PATIENT CONSENT STATEMENT

All patients included in this study provided consent to participate.

## Supporting information


Figures S1–S4.

Tables S1–S3.
