# Supplementary material for: Combined effects of high atrial septal pacing and reactive atrial antitachycardia pacing for reducing atrial fibrillation in sick sinus syndrome
Source: J Arrhythm. 2023 Jun 26;39(4):566–73. doi: 10.1002/joa3.12888 (PMC10407182; doi:10.1002/joa3.12888)
Supplement: Supplementary file 1 — Figures S1–S4. [file JOA3-39-566-s002.pdf]

Figure S1 (A)

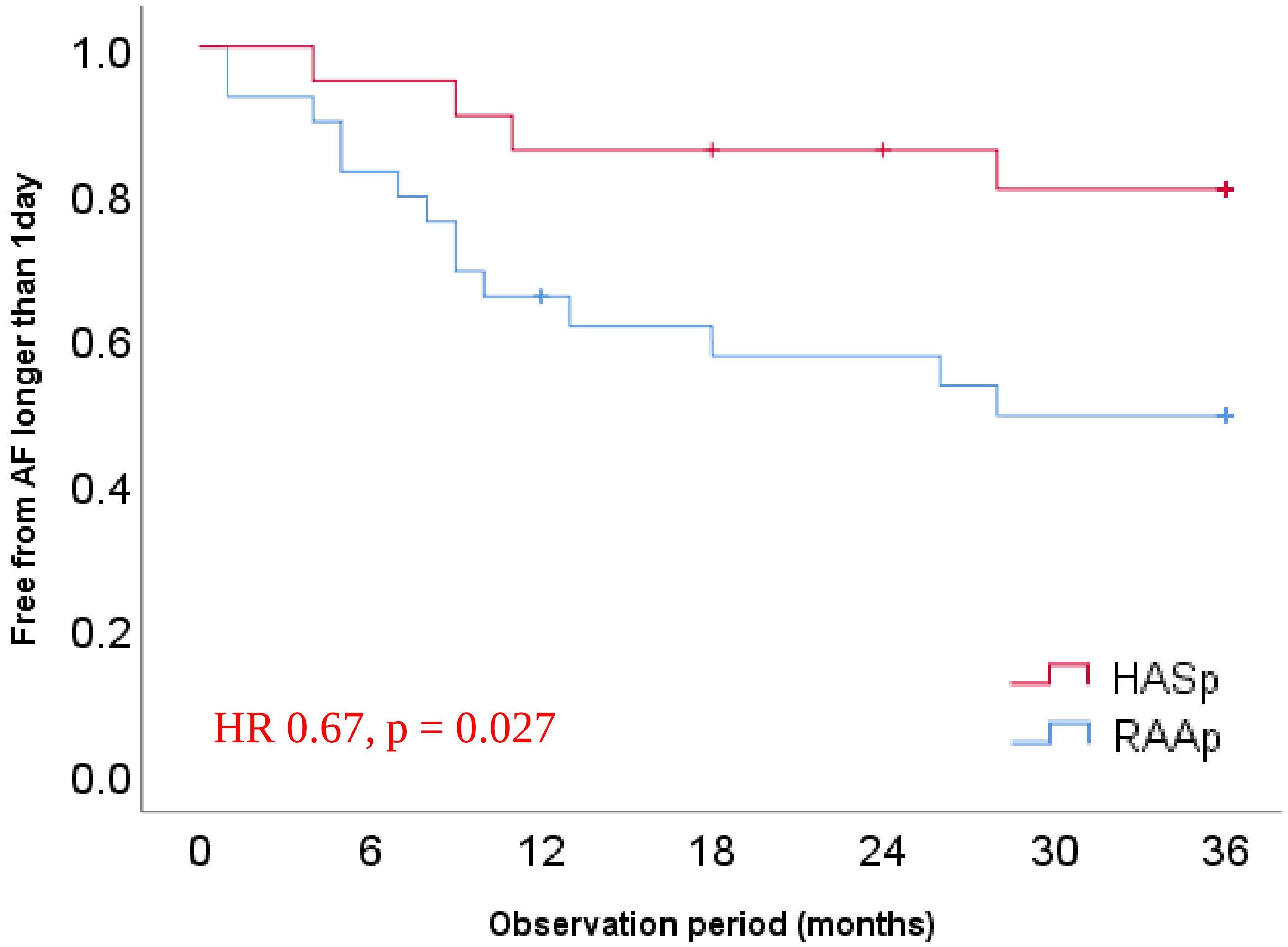

No. patients at risk (HASp, red)	21	20	18	17	16	15	15
(RAAp, blue)	29	24	16	14	14	12	12

Figure S1 (B)

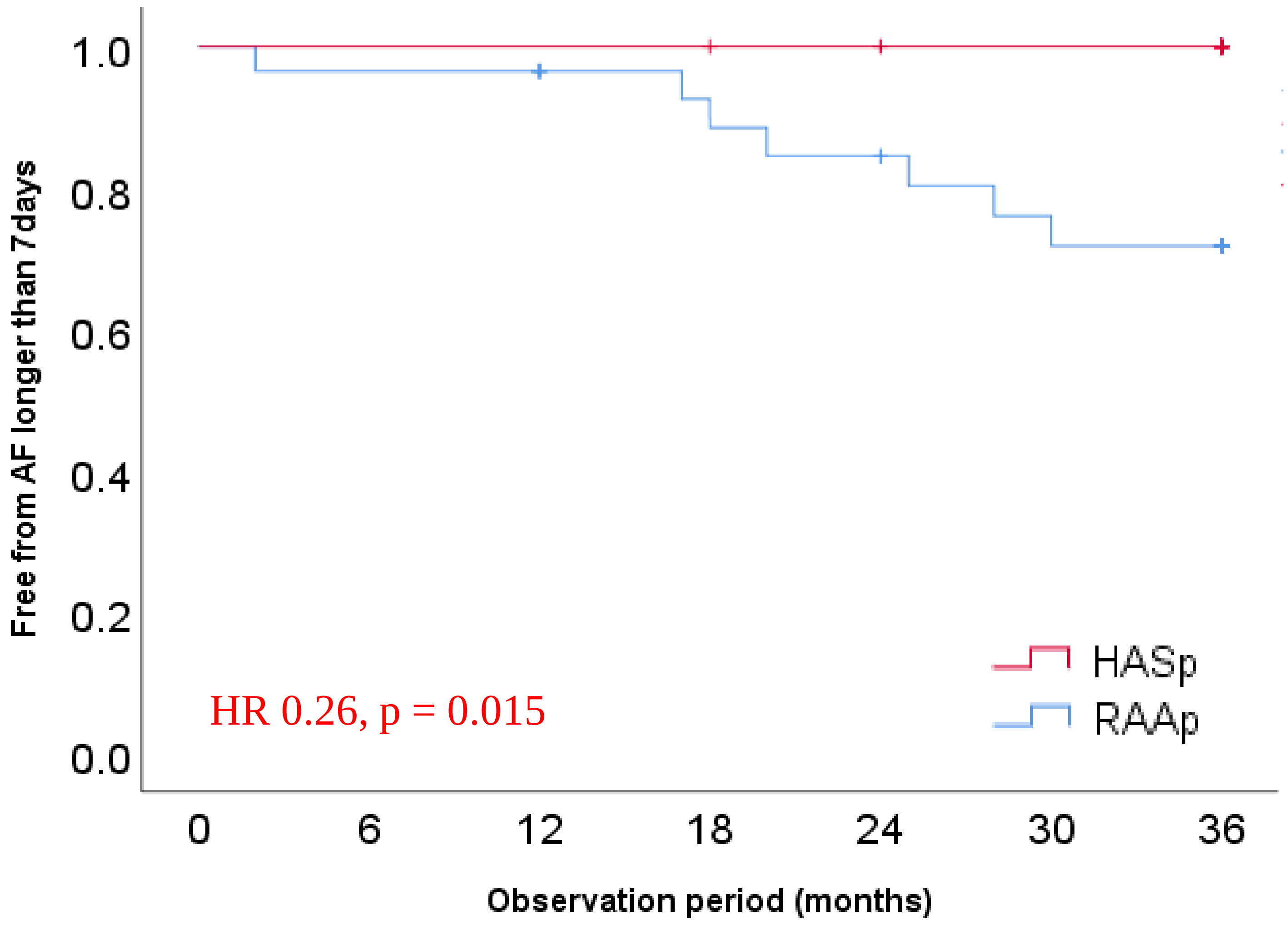

No. patients at risk	0	6	12	18	24	30	36
■ (red)	21	21	21	20	19	19	19
■ (blue)	29	28	25	22	20	17	17

Figure S1 (C)

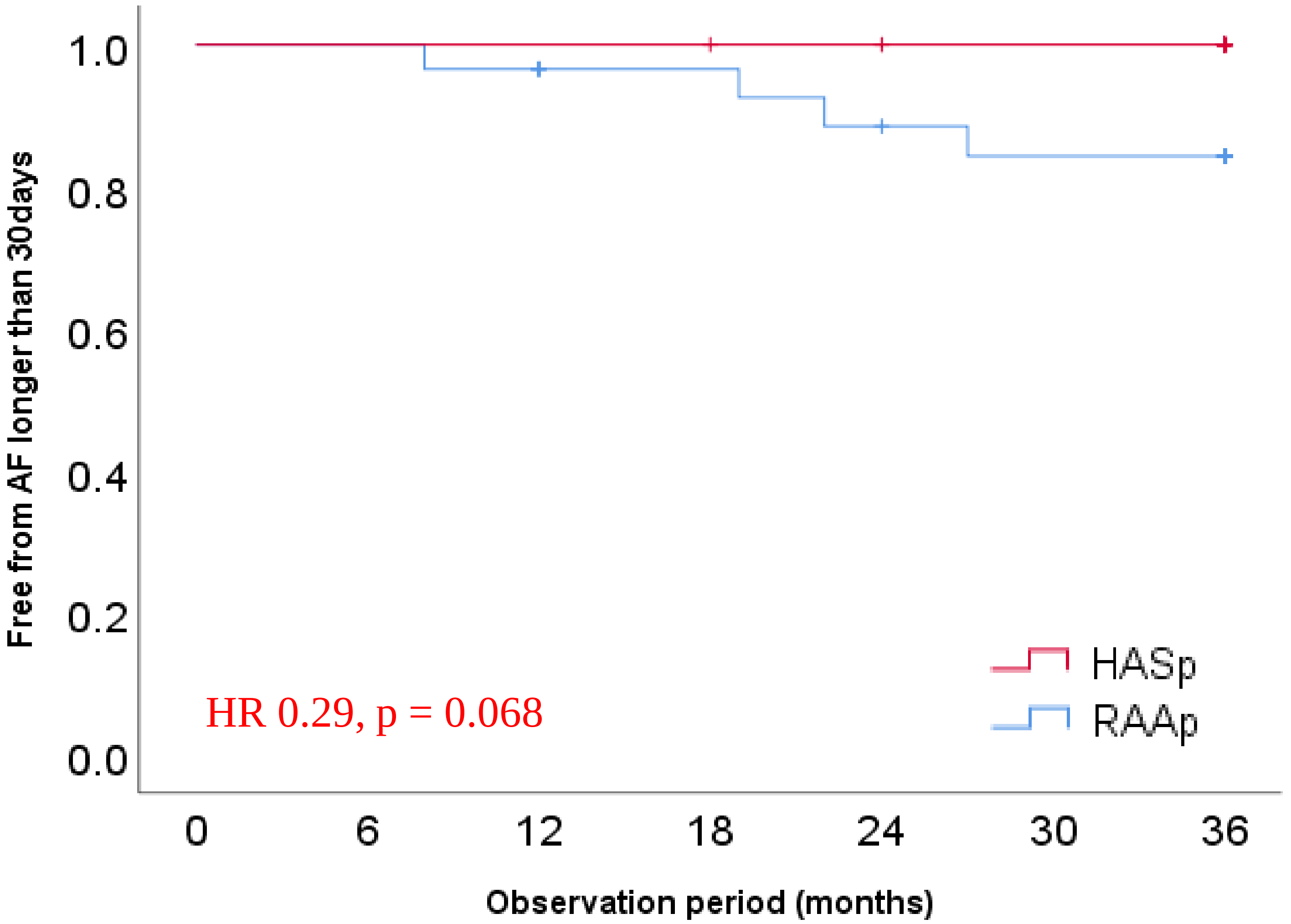

No. patients at risk							
■ (red)	21	21	21	20	19	19	19
■ (blue)	29	29	24	24	21	20	20

Figure S2 (A)

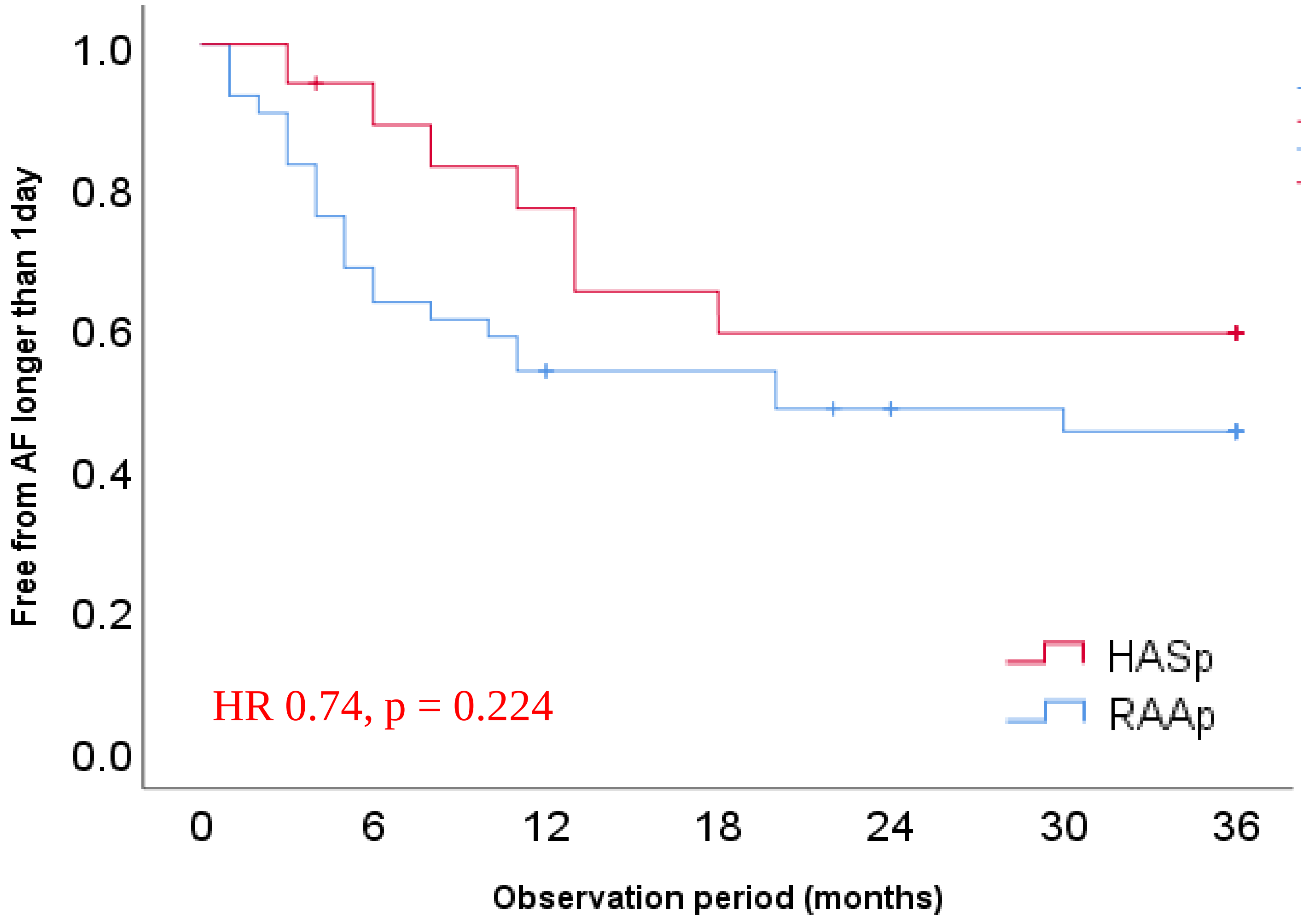

No. patients at risk							
HASp (red)	18	15	13	10	10	10	10
RAAp (blue)	41	27	20	20	15	14	14

Figure S2 (B)

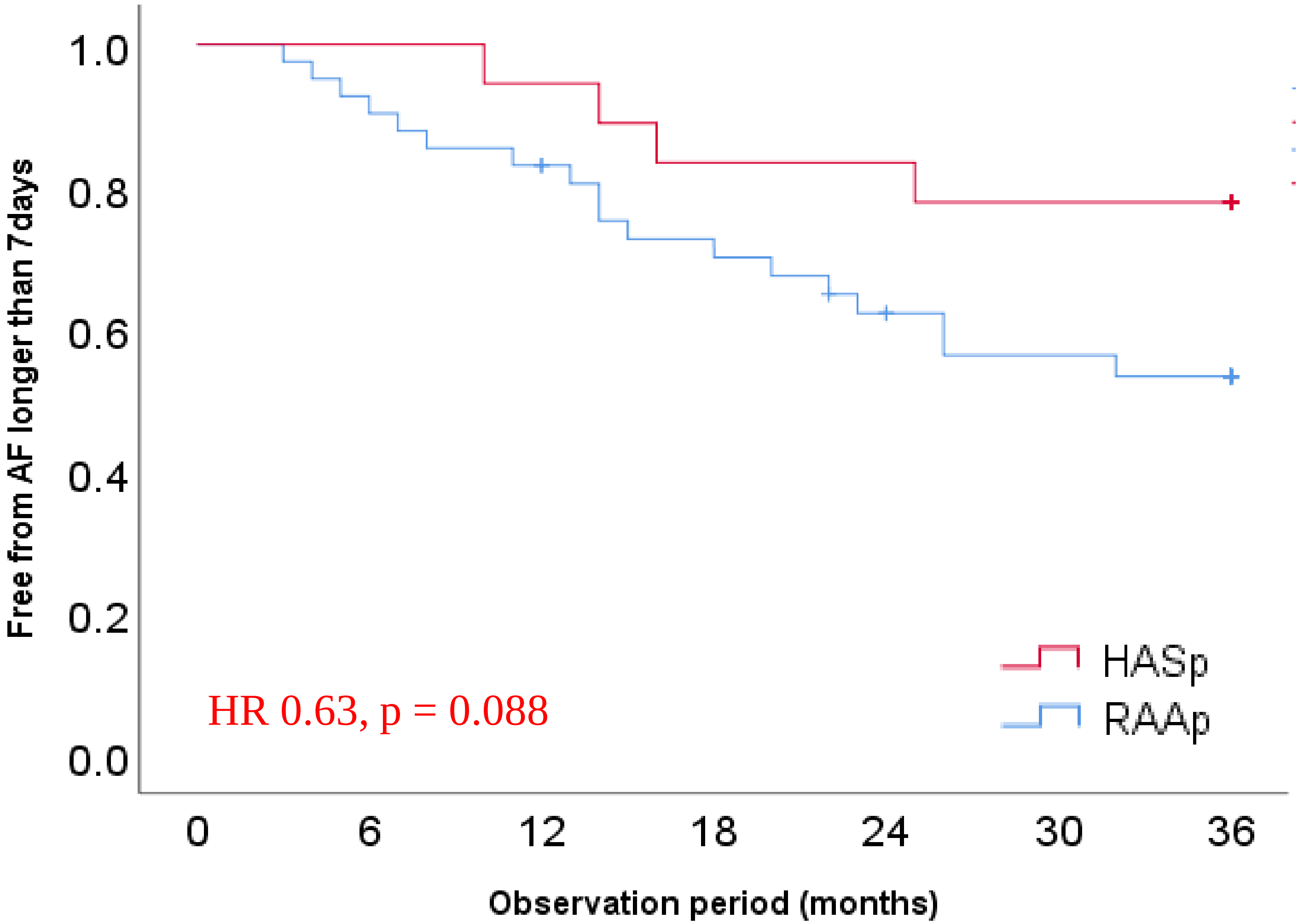

No. patients at risk							
HASp (red)	18	18	17	15	15	14	14
RAAp (blue)	41	32	27	21	21	19	18

Figure S2 (C)

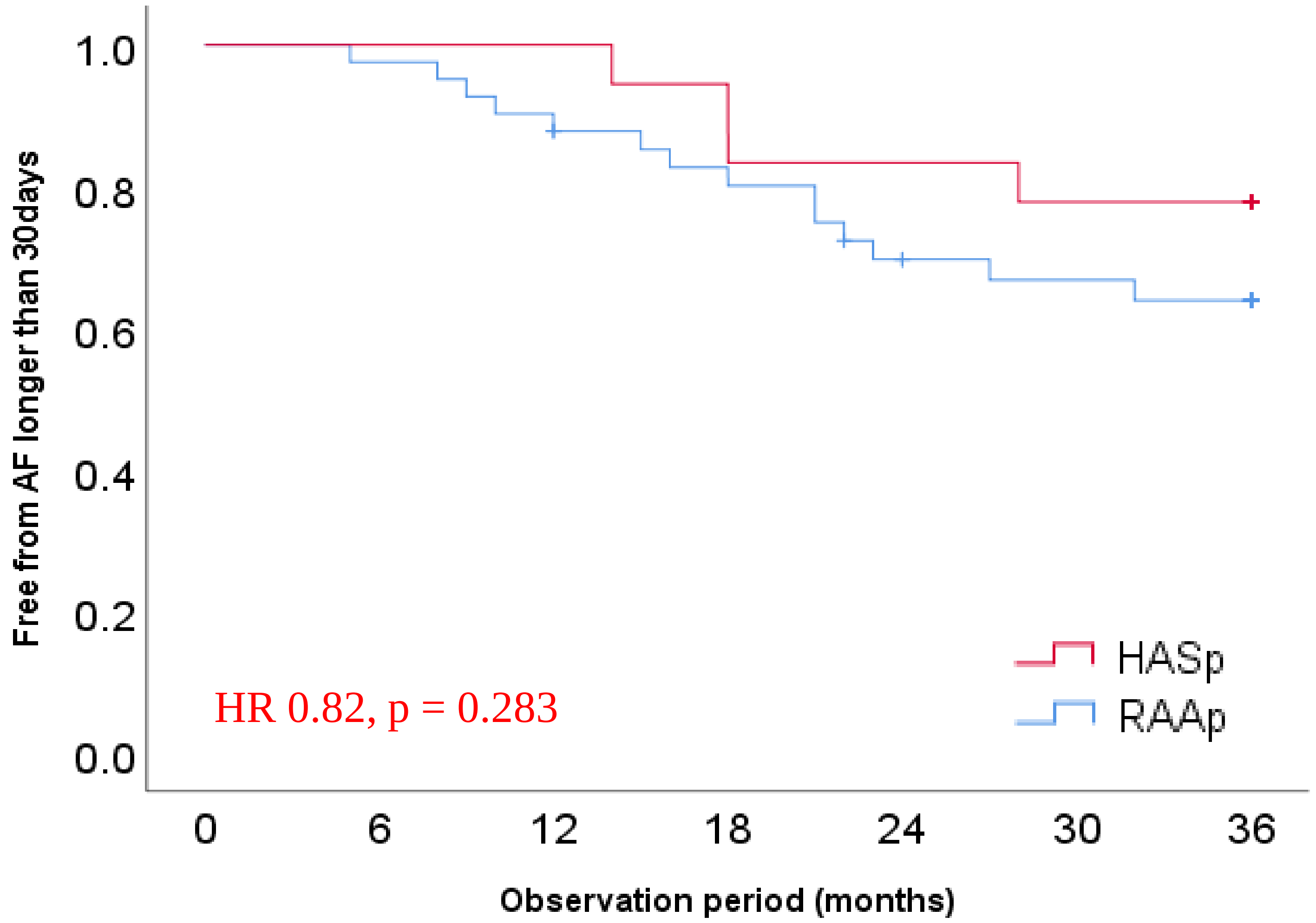

No. patients at risk							
HASp (red)	18	18	18	17	15	14	14
RAAp (blue)	41	40	36	32	26	24	23

Figure S3 (A)

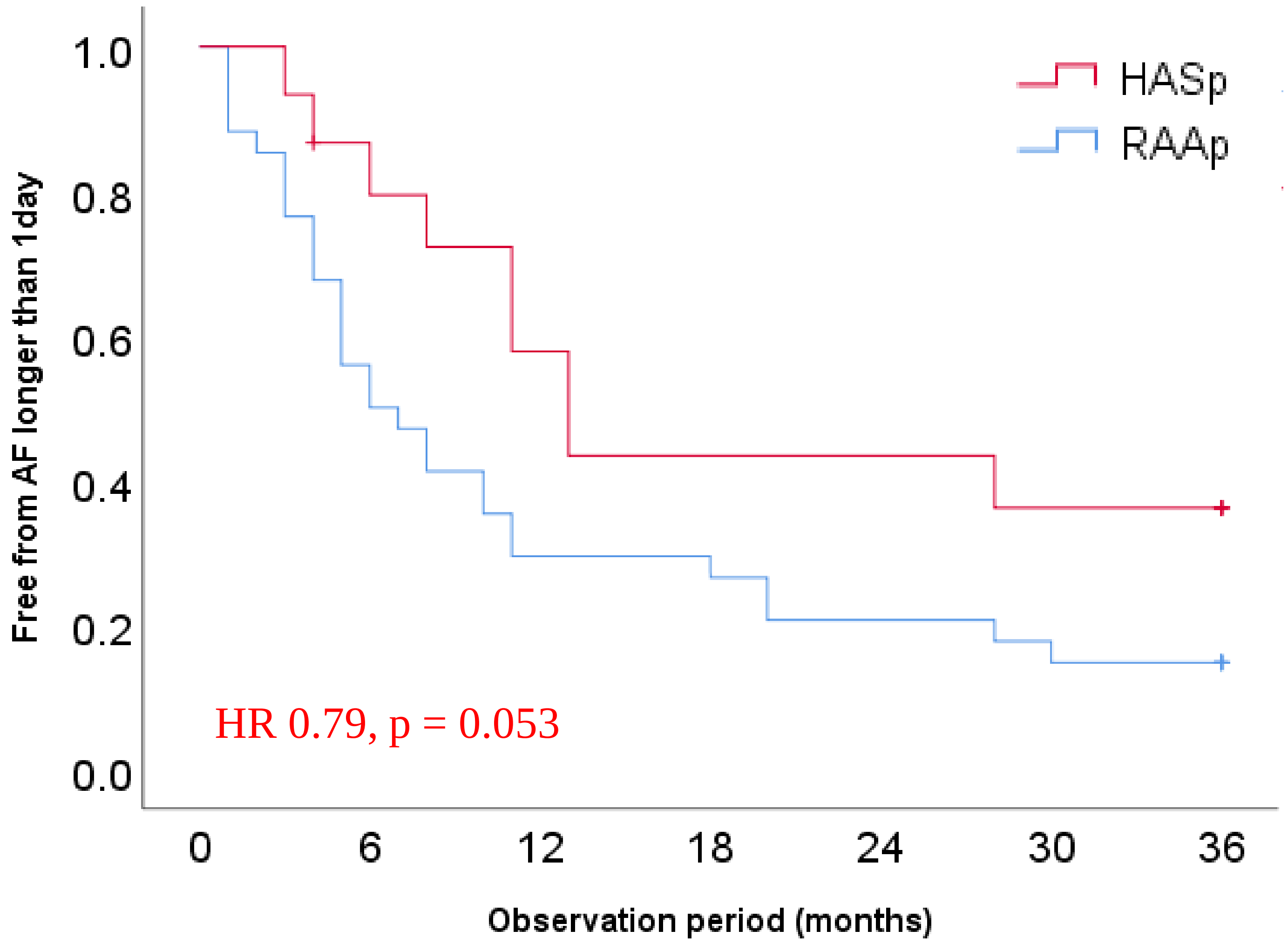

No. patients at risk							
■ (red)	15	11	8	6	6	5	5
■ (blue)	34	17	10	9	7	5	5

Figure S3 (B)

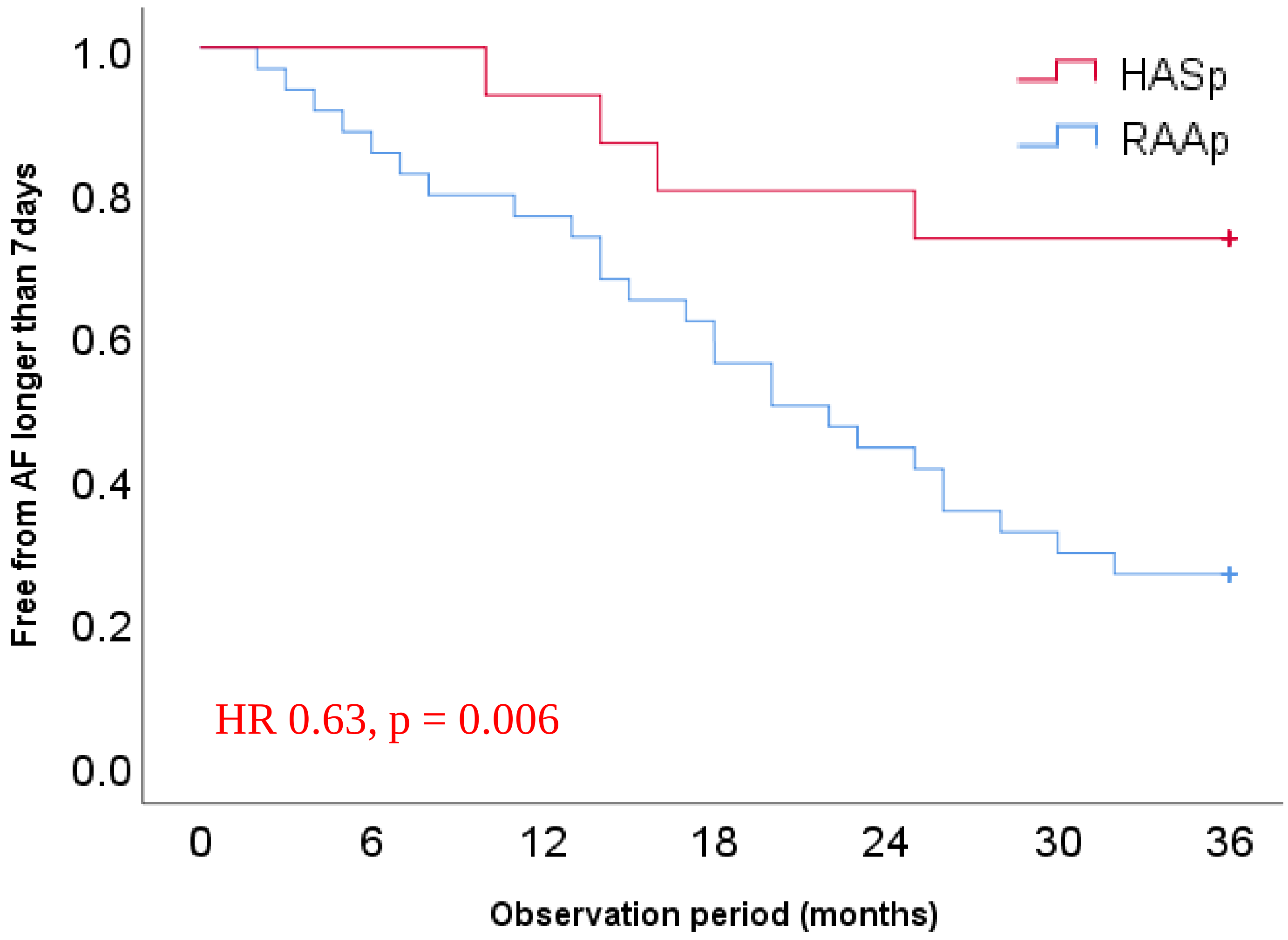

No. patients at risk							
■ (red)	15	15	14	12	12	11	11
■ (blue)	34	29	26	19	15	10	9

Figure S3 (C)

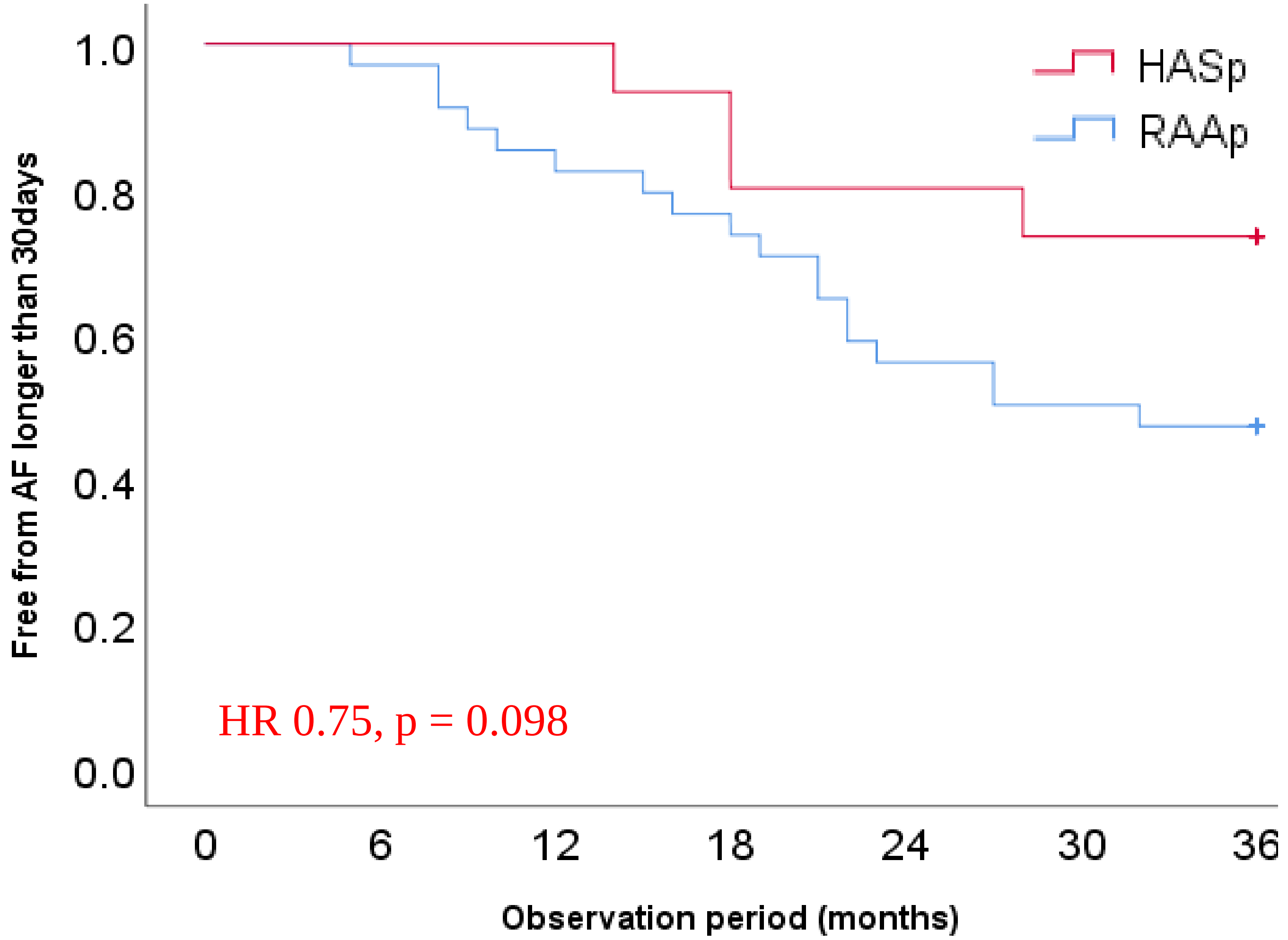

No. patients at risk							
■ (red)	15	15	14	12	12	11	11
■ (blue)	34	33	28	25	19	17	16

(A)

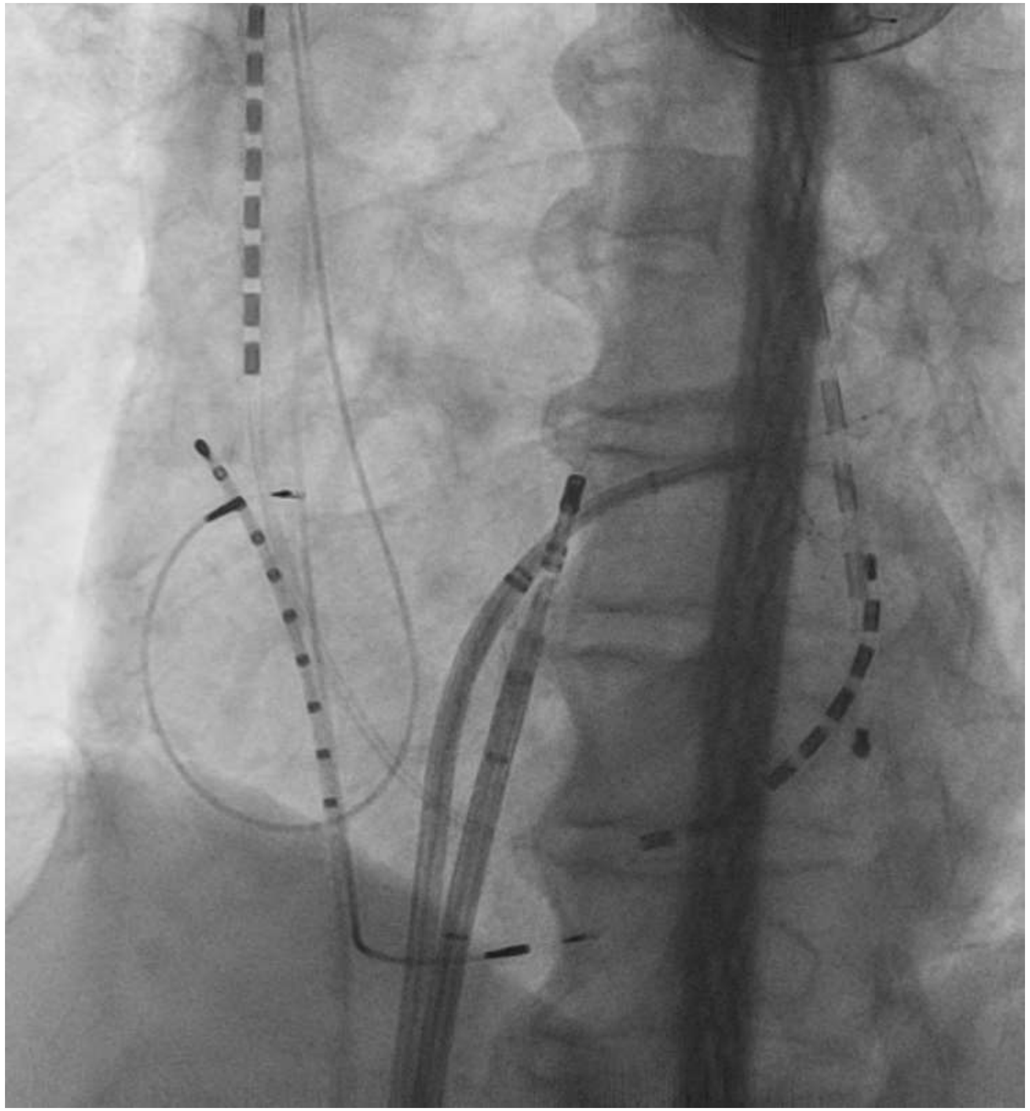

(B)

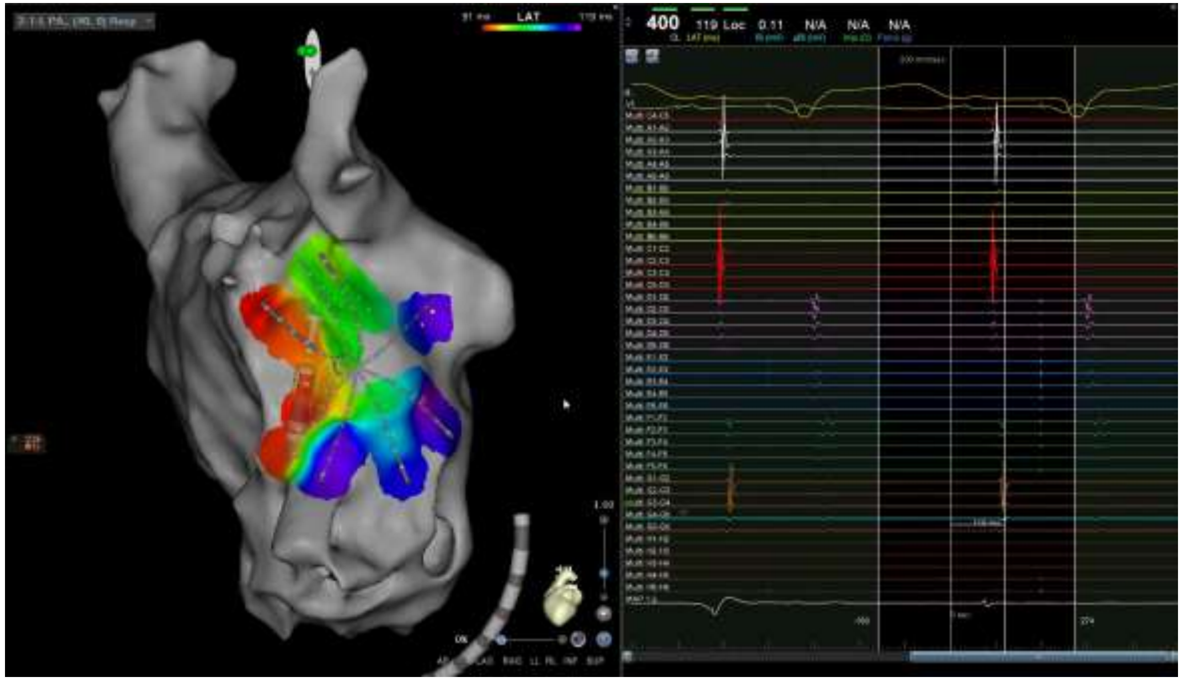

(C)

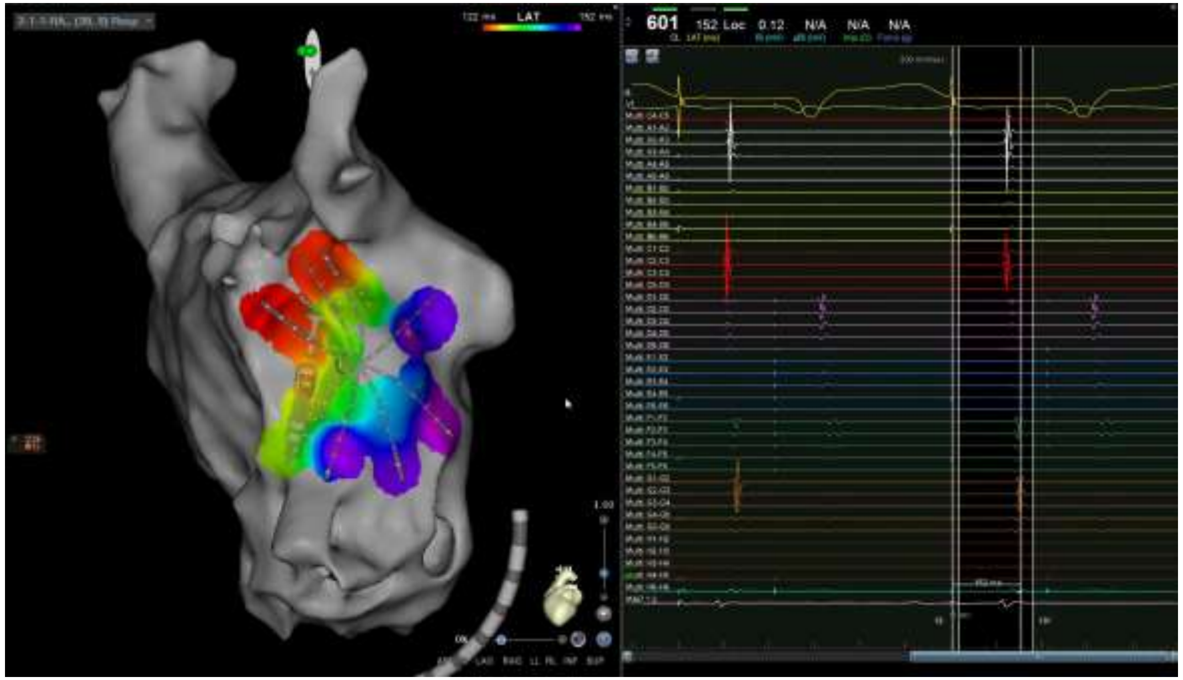

Figure S4
