# Supplementary material for: Combined effects of high atrial septal pacing and reactive atrial antitachycardia pacing for reducing atrial fibrillation in sick sinus syndrome
Source: J Arrhythm. 2023 Jun 26;39(4):566–73. doi: 10.1002/joa3.12888 (PMC10407182; doi:10.1002/joa3.12888)
Supplement: Supplementary file 2 — Tables S1–S3. [file JOA3-39-566-s001.docx]

Table S1

|  |  |  |  |
| --- | --- | --- | --- |
|  | HASp (n = 15) | RAAp (n = 34) | P-value |
| Age, mean ± SD | 80.9 ± 7.9 | 80.1 ± 6.8 | 0.711 |
| Sex, male, n (%) | 9(60.0%) | 18(52.9%) | 0.647 |
| Body mass index, mean ± SD | 21.3 ± 2.9 | 22.9 ± 3.2 | 0.508 |
| Comorbidities |  |  |  |
| History of CHF, n (%) | 5(33.3%) | 8(23.5%) | 0.344 |
| HT, n (%) | 11(73.3%) | 23(67.6%) | 0.691 |
| DM, n (%) | 4(26.7%) | 9(26.5%) | 0.989 |
| History of Stroke, n (%) | 1(6.7%) | 7(20.5%) | 0.224 |
| Medications |  |  |  |
| AAD, n (%) | 2(13.3%) | 2(5.9%) | 0.380 |
| β-adrenergic blocker, n (%) | 10(66.7%) | 20(58.8%) | 0.604 |
| ACEi or ARB, n (%) | 10(66.7%) | 15(44.1%) | 0.146 |
| Pacing characteristics |  |  |  |
| Lower rate limit (bpm), mean ± SD | 60.7 ± 2.6 | 60.0 ± 1.8 | 0.334 |
| Upper rate (bpm), mean ± SD | 124.0 ± 5.1 | 122.5 ± 5.2 | 0.367 |
| Echocardiographic parameters |  |  |  |
| Left atrial diameter (mm), mean ± SD | 40.6 ± 5.5 | 42.3 ± 6.3 | 0.390 |
| Left atrial volume index, mean ± SD | 57.4 ± 21.7 | 58.1 ± 8.6 | 0.559 |
| Ejection fraction, mean ± SD | 56.1 ± 10.9 | 58.1 ± 8.6 | 0.509 |
| P-wave characteristics |  |  |  |
| Sinus P-wave duration (ms), mean ± SD | 108.3 ± 10.9 | 104.5 ± 15.6 | 0.411 |
| Paced P-wave duration (ms), mean ± SD | 99.5 ± 7.9 | 115.2 ± 16.3 | 0.001 |

Table S2

|  |  |  |  |
| --- | --- | --- | --- |
|  | HASp | RAAp | P-value |
| Patients with more than 75% of Ap, n (%) | 21 (53.8) | 29 (41.4) | 0.212 |
| Age, mean ± SD | 81.9 ± 5.5 | 81.6 ± 5.7 | 0.884 |
| Sex, male, n (%) | 9 (42.9) | 17 (58.6) | 0.271 |
| Body mass index, mean ± SD | 22.7 ± 2.7 | 23.1 ± 3.7 | 0.714 |
| Comorbidities |  |  |  |
| History of CHF, n (%) | 4 (19.0) | 4 (13.7) | 0.544 |
| Hypertension, n (%) | 16 (76.1) | 18 (62.0) | 0.121 |
| Diabetes, n (%) | 4 (19.0) | 7 (24.1) | 0.668 |
| History of Stroke, n (%) | 2 (9.5) | 5 (17.2) | 0.438 |
| Medications |  |  |  |
| AAD, n (%) | 2 (9.5) | 5 (17.2) | 0.438 |
| β-adrenergic blocker, n (%) | 8 (38.0) | 10 (34.4) | 0.765 |
| ACEi or ARB, n (%) | 11 (52.2) | 12 (41.4) | 0.304 |
| Pacing characteristics |  |  |  |
| Lower rate limit (bpm), mean ± SD | 60.9 ± 3.0 | 60.5 ± 2.7 | 0.605 |
| Upper rate (bpm), mean ± SD | 122.9 ± 4.6 | 123.1 ± 4.7 | 0.855 |
| Echocardiographic parameters |  |  |  |
| Left atrial diameter (mm), mean ± SD | 39.0 ± 5.5 | 40.2 ± 6.0 | 0.502 |
| Left atrial volume index, mean ± SD | 51.8 ± 15.0 | 49.0 ± 18.1 | 0.573 |
| Ejection fraction, mean ± SD | 59.7 ± 5.1 | 59.4 ± 2.5 | 0.784 |
| P-wave characteristics |  |  |  |
| Sinus P-wave duration (ms), mean ± SD | 103.6 ± 11.4 | 105.7 ± 14.5 | 0.584 |
| Paced P-wave duration (ms), mean ± SD | 97.0 ± 11.1 | 116.0 ± 15.6 | ＜0.001 |

Table S3

|  |  |  |  |
| --- | --- | --- | --- |
|  | HASp | RAAp | P-value |
| Patients with less than 75% of Ap, n (%) | 18 (46.2) | 41 (58.6) | 0.212 |
| Age, mean ± SD | 81.3 ± 7.3 | 79.3 ± 7.2 | 0.336 |
| Sex, male, n (%) | 10 (55.6) | 17 (41.4) | 0.342 |
| Body mass index, mean ± SD | 21.9 ± 3.6 | 22.6 ± 2.8 | 0.464 |
| Comorbidities |  |  |  |
| History of CHF, n (%) | 3 (14.3) | 4 (9.8) | 0.344 |
| Hypertension, n (%) | 15 (83.3) | 29 (70.7) | 0.171 |
| Diabetes, n (%) | 8 (44.4) | 12 (29.2) | 0.257 |
| History of Stroke, n (%) | 2 (11.1) | 5 (12.1) | 0.747 |
| Medications |  |  |  |
| AAD, n (%) | 2 (11.1) | 6 (14.6) | 0.548 |
| β-adrenergic blocker, n (%) | 10 (55.5) | 22 (53.6) | 0.865 |
| ACEi or ARB, n (%) | 8 (44.4) | 15 (36.6) | 0.304 |
| Pacing characteristics |  |  |  |
| Lower rate limit (bpm), mean ± SD | 60.4 ± 2.2 | 60.1 ± 1.8 | 0.748 |
| Upper rate (bpm), mean ± SD | 124.4 ± 5.1 | 122.7 ± 5.0 | 0.222 |
| Echocardiographic parameters |  |  |  |
| Left atrial diameter (mm), mean ± SD | 39.4 ± 4.3 | 38.9 ± 6.6 | 0.730 |
| Left atrial volume index, mean ± SD | 47.3 ± 23.1 | 45.7 ± 15.3 | 0.344 |
| Ejection fraction, mean ± SD | 57.9 ± 11.1 | 58.1 ± 9.2 | 0.241 |
| P-wave characteristics |  |  |  |
| Sinus P-wave duration (ms), mean ± SD | 111.5 ± 11.3 | 108.0 ± 15.5 | 0.872 |
| Paced P-wave duration (ms), mean ± SD | 102.0 ± 9.3 | 116.0 ± 13.5 | ＜0.001 |

**Table S1**

Clinical characteristics of patients at the time of pacemaker implantation in those who experienced rATP.

HASp, high atrial septal pacing; RAAp, right atrial appendage pacing; AAD, anti-arrhythmic drug; ACEi, angiotensin-converting enzyme inhibitor; ARB, angiotensin receptor blocker; SD, standard deviation.

**Table S2**

Baseline characteristics between HASp and RAAp in the the high % (more than 75%) of Ap group.

HASp, high atrial septal pacing; RAAp, right atrial appendage pacing; Ap, atrial pacing; AAD, anti-arrhythmic drug; ACEi, angiotensin converting enzyme inhibitor; ARB, angiotensin receptor blocker; SD, standard deviation.

**Table S3**

Baseline characteristics between HASp and RAAp in the the low % (less than 75%) of Ap group.

HASp, high atrial septal pacing; RAAp, right atrial appendage pacing; Ap, atrial pacing; AAD, anti-arrhythmic drug; ACEi, angiotensin converting enzyme inhibitor; ARB, angiotensin receptor blocker; SD, standard deviation.
